# Should Terminal Sedation Be Expanded to Individuals Who Choose to Die Via the Voluntary Stopping of Eating and Drinking?

**DOI:** 10.1111/bioe.70127

**Published:** 2026-05-19

**Authors:** Laura Gilbertson, Dominic Wilkinson, Julian Savulescu

**Affiliations:** ^1^ Uehiro Oxford Institute University of Oxford Oxford UK; ^2^ The Royal Melbourne Hospital Melbourne Victoria Australia; ^3^ John Radcliffe Hospital Oxford UK; ^4^ Murdoch Children's Research Institute Melbourne Victoria Australia; ^5^ Centre for Biomedical Ethics, National University of Singapore Yong Loo Lin School of Medicine Singapore; ^6^ Department of Paediatrics School of Clinical Sciences, Faculty of Medicine Nursing and Health Sciences, Monash University Melbourne Victoria Australia

**Keywords:** autonomy, end of life, hastened death, right to die, terminal sedation, voluntary stopping of eating and drinking

## Abstract

The voluntary stopping of eating and drinking (VSED) is a phenomenon whereby an individual with decision‐making capacity chooses to cease eating and drinking with the intention of ending their own life. This is widely acknowledged as a lawful, albeit uncommon, end‐of‐life decision. It is now generally accepted that patients undertaking VSED should have access to appropriate palliative care, as per any other form of dying. However, it remains unclear whether terminal sedation (TS), the use of sedative drugs to treat intolerable symptoms at the end of life, should form part of this palliative care. In this paper, we explore and defend the use of TS in the management of VSED. We argue that TS is medically appropriate in the management of patients undertaking VSED who develop refractory delirium and have previously consented to TS. We further argue that, given the life expectancy window in cases of VSED, the appropriate use of sedation during this time does not hasten death and fits within the 2‐week limit applied to traditional TS. We conclude that TS is medically and ethically appropriate in the management of VSED.

## 1 Introduction

Terminal sedation (TS) describes the use of sedative drugs to alleviate suffering in dying patients, continued until the point of death [1]. Restrictions are commonly applied to the practice of TS. For example, de Graeff and Dean suggest that: symptoms should be refractory (meaning all possible treatment has failed), sedatives should be administered gradually, and death should be imminent [1]. Administered with these restrictions, although it remains somewhat controversial, TS is generally considered permissible medical practice [1, 2].

There have been calls to expand the use of TS at the end of life [3]. The traditional limits placed on TS are restrictive and there are patients who fall outside these criteria who might thereby be denied access to adequate suffering relief at the end of life. This includes patients who die via the process of voluntary stopping of eating and drinking (VSED).

During VSED, an individual with decision‐making capacity freely chooses to cease eating and drinking with the intention of hastening their own death [4]. This process is associated with significant suffering secondary to the dehydration and/or starvation resulting in death. Previous authors have argued that the suffering associated with VSED should be managed with standard palliative care, as per any other type of death [5, 6]. It remains unclear whether TS should be offered in cases of VSED. We identify only one previous study which discusses the ethics of combining VSED and TS as part of a right to self‐determined dying [7].

In this paper, we seek to consider whether TS should form part of the standard palliative care offered to patients pursuing VSED. We will answer two key questions. Firstly, is TS medically appropriate in the management of VSED? Secondly, is TS ethically appropriate in the management of VSED? Ultimately, we will argue that TS is an appropriate treatment option for the symptom of delirium in select cases of VSED. We will also argue that the life expectancy window in cases of VSED is short, and therefore TS in this setting is morally equivalent to TS in other cases of terminal illness. We consider and reject three key counter arguments.

## 2 Background

### 2.1 Terminal Sedation

Some patients are administered sedatives in the last phase of life, a practice commonly called “terminal sedation” (TS). TS is generally reserved for patients experiencing refractory suffering at the end of life, where standard palliative care measures have failed [8]. It is estimated that approximately 12%–18% of dying patients worldwide receive some level of sedation [9]. The most common indications for TS are pain, breathlessness, agitation, or delirium [9].

TS criteria typically remain restrictive across the globe, even in jurisdictions where assisted dying is legal [10]. The justification for such restrictions appears to be to minimise the ‘harms’ associated with its practice, specifically the risk of hastening death and the removal of consciousness until death. The use of TS, at least within traditional restrictive criteria, is supported by the doctrine of double effect (DDE). The DDE states that, where an action has both ‘good' and ‘bad’ consequences, it might be morally permissible to undertake the action while intending the ‘good' effect but foreseeing the ‘bad' outcome as a side effect [11]. The DDE, and its application to TS, has been widely criticised [12]. However, the doctrine continues to shape much of the ethical and legal literature concerning TS and, most pertinently, it is used to justify the restriction of TS to the final 2 weeks of life (see Section 3 for further details).

There are range of other ethical approaches applied to TS. A utilitarian framework might support its use where the benefits (alleviating intolerable suffering) outweigh the harms. Furthermore, we propose that individuals have a positive ‘right to relief of suffering’ which supports expanded access to TS as a therapeutic option (discussed further in Section 3).

There are increasing discussions around the need to expand access to sedation at the end of life [3]. Despite modern palliative care, many patients suffer at the end of life, and are denied access to sedation as a means to relieve their suffering [9]. In a separate paper we discuss the ethics of expanding access to TS beyond its traditional limits, a proposal we refer to as ‘expanded terminal sedation’ [3]. In this paper, we focus specifically on the potential expansion of TS to VSED.

### 2.2 Voluntary Stopping of Eating and Drinking

The voluntary stopping of eating and drinking (VSED), also termed the voluntary refusal of food and water, is a process by which an individual with decision‐making capacity ceases eating and drinking with the intention of hastening their own death [4]. VSED provides a means for patients to gain more control over the timing and nature of their death. A 2024 systematic review found that VSED accounts for 0.4%–1.7% of all deaths [13].

There is controversy surrounding the permissibility of VSED, including whether it constitutes an act of suicide. Jox et al. argue that a suicidal action is not always a positive act, and VSED therefore amounts to a form of ‘suicide by omission’ [14]. However, as part of normal palliative care, patients may refuse any life‐prolonging intervention, including implantable defibrillators, dialysis, intubation, or even food and water [15]. Few would consider the refusal of dialysis an act of suicide. Undeniably, however, in cases of VSED there is a clear and active intention to hasten one's own death.

The question of whether VSED constitutes an act of suicide becomes less relevant when we consider that a right to suicide exists. In most jurisdictions, adults with capacity are not legally prevented from ending their own lives, and intervention is generally only required where suicidal intent arises from a mental illness which impairs decision‐making [16]. The principle of personal autonomy respects that individuals with decision‐making capacity are free to make personal assessments about how to respond to their own suffering. It follows, therefore, that competent individuals have a right to undertake VSED.

Beyond VSED itself, the permissibility of the medical management of VSED poses a separate issue. Opponents argue that to forego artificial nutrition and to provide palliative care in cases of VSED constitutes a form of ‘assisted suicide’ [17]. Whilst suicide is usually legal, assisted suicide remains illegal in most jurisdictions [14].

However, there is a distinction between respecting a competent refusal and assisting, or even encouraging, the patient to die in this way [6]. A treating physician may attempt to respectfully discourage patients from undertaking VSED; however, if a patient is adamant in their refusal of food and water, the same physician must respect the competent refusal by not force‐feeding the patient and should offer standard palliative care, as they would for any other dying patient. Medical support for patients undertaking VSED should be adequate and proportionate to their symptoms, as per any other form of palliative care [18]. This is arguably not assisted suicide.

We acknowledge that there may be some cases in which combining these two practices could amount to assistance in suicide. Jox et al. identify two key factors which, if present, arguably classify VSED cases as assisted suicide: (a) the promise of medical assistance is instrumental to the individual's decision to pursue VSED, and (b) the physician shares, at least in part, in the individual's decision to pursue VSED (amounting to some level of encouragement) [14]. Such scenarios are not the focus of this paper. As described above, physicians need not share in a patient's intention whilst respecting their competent refusal. Furthermore, we do not propose that sedation be promised to patients at the point of undertaking VSED, but rather that it be available as a legitimate treatment option for certain patients experiencing certain symptoms. We will discuss which VSED symptoms might be an appropriate indication for sedation in Section 3.

Ultimately, the combination of VSED and palliative care is generally accepted (and we suggest would be potentially consistent with existing legal frameworks). In discussing the offering of palliative care in cases of VSED, Savulescu suggests that this might involve ‘sedation and analgesia, perhaps even so‐called “terminal sedation”’ [5]. Existing literature discussing the ethics of combining VSED and TS is scarce. In fact, we identified only one previous study, a 2024 paper by Schöne‐Seifert et al. which argues that patients undertaking VSED should be offered sedation as part of a right to self‐determined dying [7]. Further research is required to discuss the ethics of offering TS in cases of VSED, including exactly how and when it might be applied. We seek to rectify this gap.

## 3 Arguments in Favour

In considering a particular end‐of‐life option, we might assess separately whether it is medically appropriate (i.e., whether the techniques and treatments fall within a standard understanding of the goals of medicine) and whether it is ethically appropriate.

### 3.1 Is TS Medically Appropriate in the Management of VSED?

To understand if TS is medically appropriate in the management of VSED, we must first consider the symptomatic course of starvation and dehydration resulting in death. We concentrate here on patients who have chosen to completely cease both eating and drinking. Some patients embarking on VSED choose to continue reduced amounts of oral fluids. This decision may alter the trajectory of illness and prolong the dying phase. Likewise, if patients continue small amounts of feeds, symptoms of hunger persist [19]. Hence, for the purposes of discussion, we focus on those who have ceased all food and fluids (apart from small amounts of fluid for moistening the mouth and/or taking medications).

**Figure 1:**
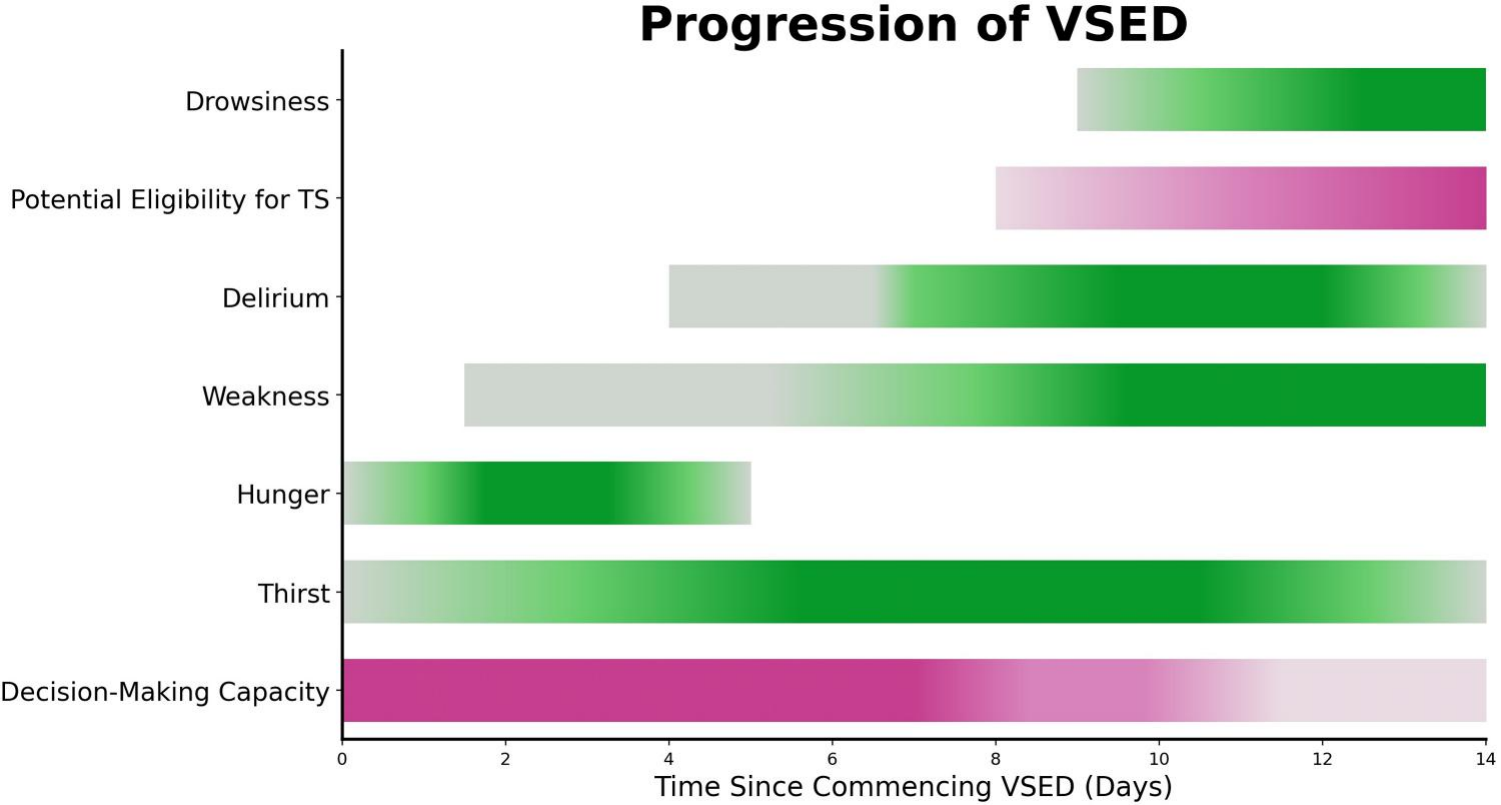
Expected progression of VSED [4].

Individuals undergoing VSED generally follow a common symptomatic course, which tends to differ only in response to the patient's hydration and nutrition status at baseline. Figure 1 describes the expected symptomatic course of VSED (adapted from Wax et al.) [4], as well as our interpretation of how decision‐making capacity and eligibility for TS might fit into this timeline.

The experience of hunger during VSED, although common initially, is relatively short‐lived and generally well tolerated requiring no specific management. Hunger usually subsides within the first 3 days of commencing VSED secondary to ketonemia [19].

By comparison, thirst is more persistent and generally more difficult to manage. The evidence‐based management of thirst and dry mouth involves mouth swabs, oral sprays, artificial saliva, and lip balm, all of which can be administered without compromising the cessation of oral fluid intake. Most individuals respond well to this treatment course, although the frequency of these interventions may be high in some patients to effectively alleviate symptoms [4].

Many patients develop delirium later in the VSED process. Delirium poses a direct threat to the quality of one's consciousness and is of particular importance when considering the appropriateness of TS in managing VSED. In the middle stages of VSED confusion levels may fluctuate. In the later stages, however, delirium can be severe and is more likely to persist until such time as the patient becomes increasingly drowsy and later dies [20].

Current delirium management in cases of VSED involves conservative reorientation and, for some patients, the use of antipsychotic medications [4]. Whilst not currently available in cases of VSED, evidence suggests that sedation is an effective intervention in managing the suffering associated with terminal delirium [21, 22].

**Figure 2:**
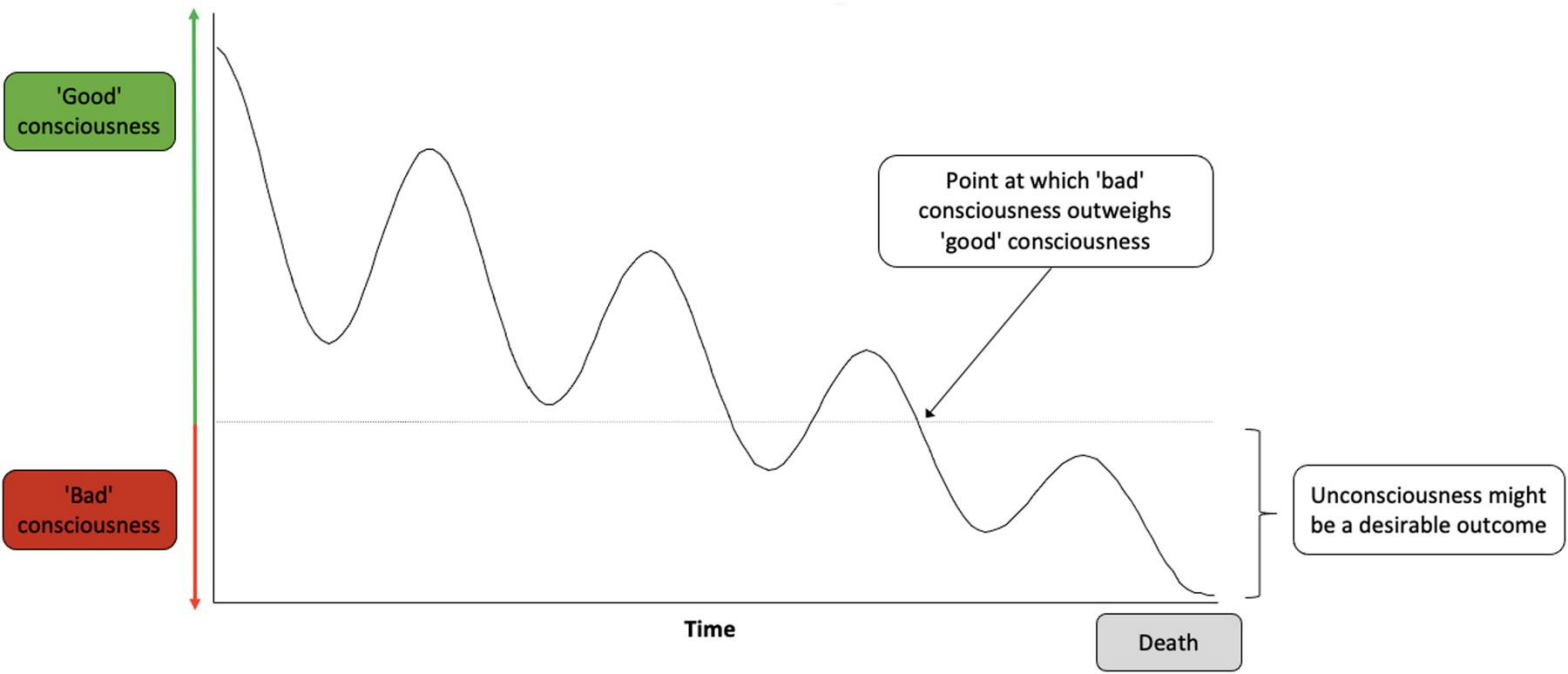
Quality of consciousness at the end of life (as taken from Gilbertson et al. 2023) [3].

Delirium is associated with significant distress. Patients experience an abrupt deterioration in cognitive and physical function, often becoming disoriented, agitated (or withdrawn), and may even experience delusions or hallucinations [22]. These symptoms make it difficult for the patient to consciously engage with their environment in a pleasant, or even meaningful, way. If the quality of one's consciousness is significantly impaired in cases of delirium, reductions in consciousness (achieved through sedation) might be desirable. Such reductions help to eliminate one's awareness of their poor quality consciousness. The desirability of unconsciousness in cases of ‘bad’ consciousness is depicted in Figure 2 below.

Recent VSED guidelines propose that sedation might be appropriate in the management of delirium that persists despite standard management and is associated with unbearable suffering [18]. In cases of so‐called ‘refractory delirium’, reductions in consciousness (through sedation) pose an ethical treatment option for the management of intolerable suffering and poor quality consciousness. This is consistent with our arguments presented above.

As a helpful framework, we propose the following criteria for VSED with TS in the setting of refractory delirium:

1. The patient is experiencing unbearable suffering.
2. The patient has lost decision‐making capacity.
3. The patient has previously stopped all fluids.
4. The patient has previously indicated that they would not wish for fluid to recommence if delirious.
5. Other measures to address confusion/distress have been attempted (or refused in advance), such as antipsychotics.

Where the above criteria are met, TS should be considered. In this context, TS might form part of the standard palliative care applied to cases of VSED. The medical appropriateness of TS in cases of refractory hunger/thirst is not the focus of this paper and provides scope for future discussion.

It is important to acknowledge the role of family members witnessing this process. Terminal thirst, hunger, and delirium are uncomfortable symptoms to observe and can prompt family requests for fluids or parenteral nutrition. Family members might also object to the use of sedation in VSED as described above. Existing literature, however, suggests that this is unlikely to be the case. Individuals initiating VSED generally do so with the explicit support of their family. These family members then commonly take on advocacy roles for the dying individual [23]. Whilst individual cases may vary, if sedation becomes an accepted part of the medical management of VSED, it is likely that family support will extend to this in most cases.

### 3.2 Is TS Ethically Appropriate in the Management of VSED?

Even where TS is medically appropriate in the management of VSED, the moral permissibility of combining these two practices poses a separate issue. As mentioned earlier, the criteria for accessing TS are commonly restricted to protect against the foreseen harms of TS (specifically, the loss of consciousness and the potential hastening of death). Importantly, most guidelines limit access to TS to the final 2 weeks of life [24, 25].

The 2‐week limit stems from the role of artificial nutrition and hydration (ANH). ANH is commonly withheld during the TS process [8]. It is assumed that if a patient has less than 2 weeks to live, withholding ANH will not shorten life, and the patient will die from their disease process. In contrast, if a patient has more than 2 weeks to live, withholding ANH will potentially hasten death as dehydration or starvation will supervene on the disease process [26]. By limiting TS to the final 2 weeks of life, sedation (without ANH) is thought to be eliminated as the cause of death and is therefore deemed morally permissible in jurisdictions that do not permit the deliberate hastening of death. Alternatively, ANH may be provided in conjunction with TS, but this raises separate questions about the length of the dying process and complications of ANH.

There are potential problems with the 2‐week limit. Firstly, and importantly, it falsely assumes that life expectancy can be predicted with accuracy. Secondly, it has the obvious consequence that dying individuals with greater than ‘2’ weeks to live are denied access to sedation even where it might be an effective means of relieving their suffering and even if they would desire this. Thirdly, the 2‐week limit is founded upon the (arguably false) assumption that the hastening of death is always impermissible.

However, irrespective of whether we agree with the 2‐week limit or not, cases of VSED involving complete cessation of food and fluids would fit within the same life expectancy window. In such cases, the standard time until death is approximately 10 to 14 days [4]. The appropriate administration of TS without ANH within this timeframe will not shorten life, and death will result from the individual's original decision to cease eating and drinking. Proportionate TS in this context does not hasten death and can be administered with the sole intention of alleviating suffering. Similar to traditional TS, the application of TS in this setting might therefore be supported by the DDE and subsequently deemed morally permissible.

Beyond the DDE, we must consider whether there is a positive moral argument in support of this practice. In an earlier paper we discuss a right to relief of suffering, defined as ‘a positive claim‐right for patients to access treatment options to relieve their suffering at the end of life’ [3]. A right to relief of suffering might support the expansion of TS to VSED as a therapeutic option. Where a VSED patient is suffering, and sedation would be a proportionate means of relieving that suffering, there would be a clear case for offering TS.

As previously mentioned, delirium is associated with significant distress and anguish, including in the context of VSED. Whilst the treating team might take reasonable steps to alleviate delirium through reorientation, companionship, or antipsychotics, if these interventions fail to effectively combat delirium (which they typically will given the standard progression of VSED), sedation might pose the next best treatment option in managing delirium. In these circumstances, access to TS would respect a right to relief of suffering.

One important consideration is to ensure that patients' wishes are being respected during this process. Doctors should have thorough discussions with their patients prior to commencing the VSED process. These discussions should include the expected symptomatic course of VSED as well as the range of treatment options available, including sedation. Where possible, patients' preferences around accessing sedation should be known prior to commencing VSED and can even be expressed in the form of an advance directive. Where patients have clearly indicated that they would wish for suffering relief (including in the form of sedation) and where the aforementioned criteria in Section 3 are met, it would be ethical to provide TS.

## 4 Arguments Against

The proposition to offer TS in cases of VSED will likely be controversial. We anticipate three key objections.

### 4.1 Incentive or Disincentive

One potential objection is that the offering of sedation (and the elimination of suffering which follows) might make individuals more likely to undertake VSED where they otherwise might not have. This argument contends that TS might lower the threshold for initiating VSED and make it more ‘enticing’ to individuals wishing to end their life. Where the potential for sedation is instrumental to the individual's decision to pursue VSED, this might be classified as assisted suicide (as per the arguments presented by Jox et al.) [14].

However, we believe that this harm can be reasonably mitigated through a thorough pre‐assessment of individuals requesting VSED. Prior to initiating physician involvement in the VSED process, physicians should seek to confirm that the individual (a) has decision‐making capacity, and (b) expresses a genuine intention to end their life. This pre‐assessment should also seek to confirm that the individual is fully informed, their decision is voluntary, their decision is consistent with their known values, and that the individual is free from mental illness compromising their decision.

The VSED process requires significant strength of character (motivated by a genuine desire to end one's life); this is true irrespective of the offering of TS. Furthermore, the experience of thirst and hunger, whilst able to be managed with other palliative care measures, are still arguably unpleasant human experiences which a competent individual only weathers where they have a genuine desire to end their life.

It is further important to emphasise that TS is positioned in this paper as a treatment option for some of the symptoms associated with VSED, namely refractory delirium, but not all. We do not claim that sedation should be enacted the moment that VSED is initiated. It is our view that TS is a proportionate treatment option for some of the symptoms associated with VSED, as per a right to suffering relief at the end of life and as is consistent with standard palliative care. Using the very distinction presented by Jox et al., this would not be classified as assisted suicide.

### 4.2 Change of Mind

Another objection we anticipate concerns the reversibility of VSED. There is evidence that during the course of VSED some individuals change their mind and make the decision to recommence eating and drinking and not to hasten their death [4]. Critics might argue that to sedate a patient to unconsciousness eliminates the possibility of a patient communicating a change of mind and therefore might commit individuals to death via VSED where they otherwise would have made the decision to continue living.

However, it is important here to recall our focus in the proposal. Since we have suggested restricting TS to cases of refractory delirium (and provided conditions for this), providing TS would not prevent any patients with capacity from changing their mind and deciding to discontinue VSED.

Rather, the question is how to respond to patients who might make requests for food and water after they have lost decision‐making capacity. Case studies suggest that individuals with delirium during VSED sometimes forget about their prior intention to cease eating and drinking [4]. It is unclear how often this occurs, but requests for food and water at this stage are not necessarily complied with [4]. Whilst this may be distressing to caregivers (and treating physicians), obeying with such demands would potentially protract the dying process and contradict the patient's clear prior intention to hasten death [4].

It is recommended to discuss with patients in advance of commencing VSED how they would like health professionals to respond if, during the course of the ensuing days, they make requests for food/fluids but appear to have lost understanding and awareness of their circumstances. If the patient would wish for fluids to continue to be withheld (and they have separately consented to this), sedation may be an appropriate response. Of course, if at any time the degree of delirium and/or decision‐making capacity is uncertain, the patient's case may be referred for urgent second opinion and/or ethics review.

### 4.3 Patients Who Lack Decision‐Making Capacity

A third potential objection concerns whether this proposal might inadvertently progress to the ‘non‐voluntary stopping of eating and drinking’; in other words, the administration of TS to patients who lack decision‐making capacity who have naturally ceased eating and drinking at the end of life. These are typically patients with advanced dementia (or other progressive neurological conditions) who experience mechanical issues with eating and drinking.

Whilst such individuals are not the focus of this paper, we acknowledge that our proposal might eventually, and naturally, extend to this cohort. It is not standard practice to forcefully feed/hydrate patients with advanced dementia who naturally cease eating and drinking [27]. This decision is made on ethical grounds (i.e., that life cannot be perpetually prolonged) as well as an acknowledgement that force–feeding will likely do more harm than good (including the risk of aspiration, agitation, or need for restraint) [27].

Once these patients have naturally ceased eating and drinking (and the decision is made to forego ANH), they will inevitably die from the starvation and/or dehydration resulting in death. Compared to VSED, life expectancy is generally shorter and symptoms may be less severe [28]. Nonetheless, where these patients suffer intolerably, they have a right to adequate suffering relief, including TS, as per any other dying patient. We acknowledge that the potential expansion of TS to patients who lack decision‐making capacity who cease eating and drinking is controversial and will require further research.

## 5 Conclusion

In this paper, we have considered the ethics of applying terminal sedation (TS) to the management of the voluntary stopping of eating and drinking (VSED). Whilst both practices are well described in the literature, they have often been viewed as distinct entities, without thorough consideration of how TS might form part of the management of dying in cases of VSED.

Proportionate palliative care is accepted in cases of VSED [5], yet it previously remained unclear whether TS should form part of this standard palliative care. We argue that TS is a medically and ethically appropriate treatment option for managing refractory delirium in VSED patients. Delirium poses a direct threat to the level and quality of one's consciousness, and subsequent reductions in consciousness (including through sedation) are therefore morally permissible. Furthermore, the life expectancy window in cases of VSED fits within the 2‐week limit applied to traditional TS. Subsequently, proportionate TS in this setting does not hasten death and is morally equivalent to TS in other cases of terminal illness.

The potential expansion of TS to VSED is important for two key reasons. Firstly, it forms part of the move to expand access to sedation at the end of life and to ensure that individuals who would benefit from sedation are not left to suffer unnecessarily. Secondly, it increases the range of treatment options available to VSED patients and increases the likelihood of this being a ‘good’ death. Although not the focus of this paper, the range of options available to VSED patients might even extend to assisted dying in jurisdictions where this is legal (as was recently successfully carried out by a VSED patient in Oregon in the United States) [29]. The ethics of combining VSED and assisted dying provides scope for further research.

Ultimately, sedation should form part of the palliative care standardly available in cases of VSED. Individuals undergoing VSED who are fully informed and consent in advance to sedation should have a right to do so when it is medically appropriate, and at the point at which they appear to have irreversibly lost capacity. This is true of any other dying patient. VSED patients have a right to a good death.

## Conflicts of Interest

J.S. is a Bioethics Committee consultant for Bayer and an Advisory Panel member for the Hevolution Foundation (2022‐). The other authors declare no conflicts of interest.

## Description of How the Paper Was Written

L.G. performed the literature search, had the initial idea and initial ethical analysis, wrote the first draft of the paper, and revised for publication. D.W. and J.S. supervised the research, contributed to ethical analysis, and edited the paper.

## Data Availability

The authors have nothing to report.
